# Risk of clear-cell adenocarcinoma of the vagina and cervix among US women with potential exposure to diethylstilbestrol in utero

**DOI:** 10.1007/s10552-022-01598-3

**Published:** 2022-06-29

**Authors:** Mary C. White, Hannah K. Weir, Ashwini V. Soman, Lucy A. Peipins, Trevor D. Thompson

**Affiliations:** 1 Division of Cancer Prevention and Control, Centers for Disease Control and Prevention, Atlanta, GA, USA; 2 Cyberdata Technologies, Inc., Herndon, VA, USA; 3 Epidemiology and Applied Research Branch, Division of Cancer Prevention and Control, Centers for Disease Control and Prevention, 4770 Buford Highway NE, MS S107-4, Chamblee, GA 30341, USA

**Keywords:** Diethylstilbestrol, DES, Gynecologic cancer, Cancer registries, Prenatal exposure, Cancer surveillance

## Abstract

**Purpose:**

Women exposed to diethylstilbestrol (DES) in utero were at elevated risk of clear-cell adenocarcinoma of the vagina and cervix (CCA) as young women. Previous research suggested that this elevated risk of CCA may persist into adulthood. We extended a published analysis to measure CCA risk as these women aged.

**Methods:**

Standardized incidence ratios (SIR) compared CCA risk among women born from 1947 through 1971 (the DES-era) to CCA risk among the comparison group of women born prior to 1947, using registry data that covered the US population.

**Results:**

Incidence rates of CCA among both cohorts increased with age. Among the DES-era birth cohort, higher rates of CCA were observed across all age groups except 55–59 years. SIR estimates had wide confidence intervals that often included the null value.

**Conclusions:**

Results are consistent with prior research and suggest an elevated risk of CCA in midlife and at older ages among women exposed in utero to DES. These results highlight unresolved issues regarding cancer risk among aging DES daughters and appropriate screening guidance. The examination of population-based cancer surveillance data may be a useful tool for monitoring trends in the incidence of other rare cancers over time among specific birth cohorts.

## Introduction

Women whose mothers took diethylstilbestrol (DES) while pregnant during the middle of the last century had an elevated risk of clear-cell adenocarcinoma of the vagina and cervix (CCA) as young women [1, 2]. The upper age limit for elevated CCA risk among these women, if any, remains unknown. A study of over 12,000 DES-exposed women in the Netherlands reported an elevated CCA risk among women aged ≥ 40 years, based on two cases observed (between 40 and 49 years) and 0.1 case expected [3]. A study at the University of Chicago of CCA registry cases from 1971 to 2014 reported a peak in incidence of DES-related CCA at age 20 years and a second smaller peak at age 42 years [4]. In a previous publication using combined registry data covering the US population up through 2006, we reported a second peak in CCA risk after age 40 among women born between 1947 and 1971, a period of pre-natal DES use, compared to women born before or after this period [5]. By repeating the analysis for this birth cohort more than a decade later, we aimed to measure the risk of CCA among these women as they aged.

## Methods

### Incidence and population data

Cancer incidence data came from two federal cancer surveillance programs: the Centers for Disease Control and Prevention (CDC)’s National Program of Cancer Registries (NPCR) and the National Cancer Institute (NCI)’s Surveillance, Epidemiology and End Results (SEER) Program, which together cover 100% of the US population [6]. Patient demographic and tumor characteristics are collected and reported to CDC and NCI according to standards established by the North American Association of Central Cancer Registries [7]. Data from both NPCR and SEER registries meeting high quality standards for diagnosis years 1998 through 2018 were combined in the United States Cancer Statistics (USCS) database (44 cancer registries, covering 91.4% of the US population) [8]. County level population estimates were produced by the US Census Bureau with further refinements by NCI [9].

### Analysis

We followed the methods described previously [5], with a few modifications. Cases of invasive clear-cell adenoma carcinomas of the cervix or vagina (CCA) were identified in women aged 40–69 years using ICD-O-3 morphology code 8310/3 and site codes C52 and C53, respectively [10]. Data for women born from 1947 through 1971 (the DES-era birth cohort) were selected to correspond with the period when pregnant women were most frequently prescribed DES in the United States. Supplementary Figure 1 illustrates the progression of this cohort of women by age, calendar year, and the age groups covered by each registry data source.

The measure of association was a standardized incidence ratio. We compared observed case counts among the DES-era cohort to expected case counts for women not exposed in utero to DES. SEER-9 data for years 1975 to 2011 were used to estimate incidence rates among women born prior to 1947 in each 5-year age group (Table 1). SEER*Stat was used to estimate the age-specific incidence rates. The incidence rates of CCA in these comparison groups were then multiplied by the age-specific estimates of the population at risk among the DES-era birth cohort for the expected counts. An observed to expected (O/E) ratio was calculated for each 5-year age group. The corresponding 95% confidence intervals were calculated based on exact intervals for the binomial probability of CCA occurrence among the DES-era birth cohort.

## Results

Among women in the comparison cohort, those born before 1947, CCA incidence rates tended to increase with increasing age (Table 1). CCA incidence rates among women in the DES-era birth cohort also tended to increase with age, regardless of which cancer registry source was used to calculate incidence rates. However, age-specific incidence rates for CCA were generally higher for the DES-era birth cohort than for the comparison cohort.

Among the DES-era birth cohort, elevated risk was observed across all age groups except women aged 55–59 years (Table 2). The ratios between observed and expected cases were largest for women aged 40–44 years and 45–49 years. Limited case numbers may account for the wide 95% confidence intervals, sometimes ranging from no excess risk to substantial risk, reflecting uncertainty in the magnitude of excess risk. The 95% confidence intervals were wider for rate ratios based on SEER-9 data than for USCS data because of the relative sizes of these databases.

## Discussion

In a previous analysis based on data through 2006, we reported an elevated risk of CCA among women born in the DES-era at ages 40–44 years, 45–49 years, and 50–54 years [5]. The present study extends the earlier analysis by examining data through 2018 and providing new information about CCA risk at ages 55–59 years, 60–64 years, and 65–69 years. CCA risk appeared to increase among the DES-era birth cohort at ages 60–64 years and 65–69 years. These additional years of data also increased the size of the observed population at risk. We observed elevated rate ratios for women at ages 40–44 years, 45–49 years, and 50–54 years that were similar in magnitude to those based on fewer women years and previously reported [5].

Although our study was based on cancer registry data that covered 91.4% of the US population, the infrequency of CAA resulted in measures of rate ratios with wide confidence intervals. We had no information on hysterectomy status, and failure to adjust for hysterectomy status can lead to underestimates of cervical cancer incidence rates [11]. In this study, clear-cell adenocarcinoma of the cervix comprised 82% of the CCA cases. The prevalence of hysterectomy at ages 60–69 years among women born during the DES-era was about 30–40% [11], roughly similar to that reported among older women born earlier [12, 13]; lack of adjustment for hysterectomy status was unlikely to be a major source of bias. Other unidentified risk factors may explain some of the differences seen between the comparison cohort and the DES-era birth cohort. Previous studies of DES-exposed women found no relationship between CCA and oral contraception, pregnancy, or human papillomavirus [14, 15].

Our measure of exposure was crude because it was based on only year of birth. We do not know who may have been exposed in utero to DES. Although the total number of women prescribed DES during pregnancy has been estimated to be between 2 and 4 million [4, 16], this is a small fraction of the number who gave birth between 1947 and 1971. Treating all women born during this period as potentially exposed creates substantial misclassification of exposure and could dilute or mask the true measure of risk among those women with actual in utero exposure to DES.

Our study contributes to a limited body of evidence regarding potential cancer risk among aging DES daughters. Current cervical cancer screening guidelines and recommendations for average risk women, including an age to stop screening, do not apply to women exposed in utero to DES [17]. The National Cancer Institute has noted the absence of published guidelines on medical examinations and screening from major organizations to address the specific needs of older DES daughters [18]. Based on the experience of the NCI DES combined cohort, Troisi et al. concluded that in utero exposure to DES appeared to be associated with life-long increased risk, but small absolute risk, of all lower genital tract cancers [19].

A unique strength of the present study was the inclusion of nearly all CCA cases in the United States during the period of interest. The risk of rare cancers like CCA can be difficult to study. Case reporting to the CCA registry at the University of Chicago declined after enactment of the HIPPA law in 2003, just as many DES daughters were entering midlife [4]. The NCI DES combined cohort includes women with documented in utero exposure to DES, but the cohort may be insufficiently large to monitor rare CCA events; a follow-up published online in 2017 reported four observed CCA cases (at ages 22–39 years) among 4822 exposed women compared with 0.15 expected [19].

Our study approach offers an alternative to assess the risk of CCA at older ages among the generation of women born during the DES-era, a period when mothers were prescribed prenatal DES. Our results suggest the possibility of an elevated risk of CCA in midlife and at older ages among women exposed in utero to DES. As more women born during the DES-era move into midlife and beyond, estimates of CCA risk for women aged 50–69 years could be based on more women years, and measures of CCA risk at ages 70 years and older will be possible. The examination of population-based cancer surveillance data may be a useful tool for monitoring trends in the incidence of this or other rare cancers among specific birth cohorts.

## Supplementary Material

Supplementary Figure 1

## Figures and Tables

**Table 1:** Age-specific annual incidence rates of clear-cell adenocarcinoma of the cervix and vagina by birth cohort

Age at diagnosis (years)	Comparison birth cohort (born before 1947)			DES-era birth cohort (born between 1947 and 1971)			
	Year of birth	Year of diagnosis	CCA rate SEER-9	Year of birth	Year of diagnosis	CCA rate SEER-9	CCA rate USCS
65–69	1906–1946	1975–2011	1.86	1947–1953	2016–2018	2.86	2.62
60–64	1911–1946	1975–2006	0.86	1947–1958	2011–2018	1.50	1.56
55–59	1916–1946	1975–2001	1.28	1947–1963	2006–2018	1.48	1.23
50–54	1921–1946	1975–1996	0.63	1947–1968	2001–2018	1.15	1.25
45–49	1926–1946	1975–1991	0.30	1947–1971	1996^a^-2016	0.74	0.98
40–44	1931–1946	1975–1986	0.28	1947–1971	1991^a^-2011	1.23	0.95

Rates are per 1,000,000 women years

*CCA* clear-cell adenocarcinoma of the cervix and vagina, *SEER* Surveillance, Epidemiology and End Results, *USCS* U.S. Cancer Statistics

a USCS rates for year 1998 onwards

**Table 2:** Observed and expected case counts and observed to expected incidence ratios of clear-cell adenocarcinoma of the cervix and vagina among women in the DES-era birth cohort by data source and age at diagnosis

Age at diagnosis (years)	SEER-9				USCS			
	Women years at risk	Observed (O) cases	Expected (E) cases^a^	O/E (95% CI)	Women years at risk	Observed (O) cases	Expected (E) cases^a^	O/E (95% CI)
65–69	2,451,740	7	4.56	1.54 (0.57, 3.54)	24,453,581	64	45.48	1.41 (0.91, 2.22)
60–64	7,314,458	11	6.31	1.74 (0.72, 4.13)	71,908,912	112	62.02	1.81 (1.03, 3.41)
55–59	12,834,433	19	16.47	1.15 (0.58, 2.30)	125,146,653	154	160.56	0.96 (0.59, 1.64)
50–54	18,325,151	21	11.61	1.81 (0.77, 4.72)	177,275,003	217	112.29	1.93 (0.96, 4.53)
45–49	21,595,103	16	6.56	2.44 (0.70, 13.75)	189,222,133	186	57.52	3.23 (1.09, 15.82)
40–44	21,922,492	27	6.07	4.45 (1.12, 38.58)	142,523,588	135	39.48	3.42 (0.93, 28.52)

Women born between 1947 and 1971

*SEER* Surveillance, Epidemiology and End Results, *USCS* U.S. Cancer Statistics

a Expected cases obtained by applying the incidence rate for clear-cell adenocarcinoma among the comparison birth cohort to the age-specific population of women of the DES-era birth cohort in SEER and USCS data for the respective time period

## Data Availability

The data used in this study from the U.S. Cancer Statistics public-use databases are provided and analyzed through SEER*Stat software, which is distributed by the NCI’s SEER Program. As the databases contain data from both CDC’s NPCR and NCI’s SEER program, data-use agreements from both programs must be signed. Additional information on how to access these data are available at: https://www.cdc.gov/cancer/uscs/public-use/obtain-data.htm.
