# Supplementary figures and images for: Risk of clear-cell adenocarcinoma of the vagina and cervix among US women with potential exposure to diethylstilbestrol in utero
Source: Cancer Causes Control. Author manuscript; Available in PMC 2023 Aug 1. (PMC9377316; doi:10.1007/s10552-022-01598-3)

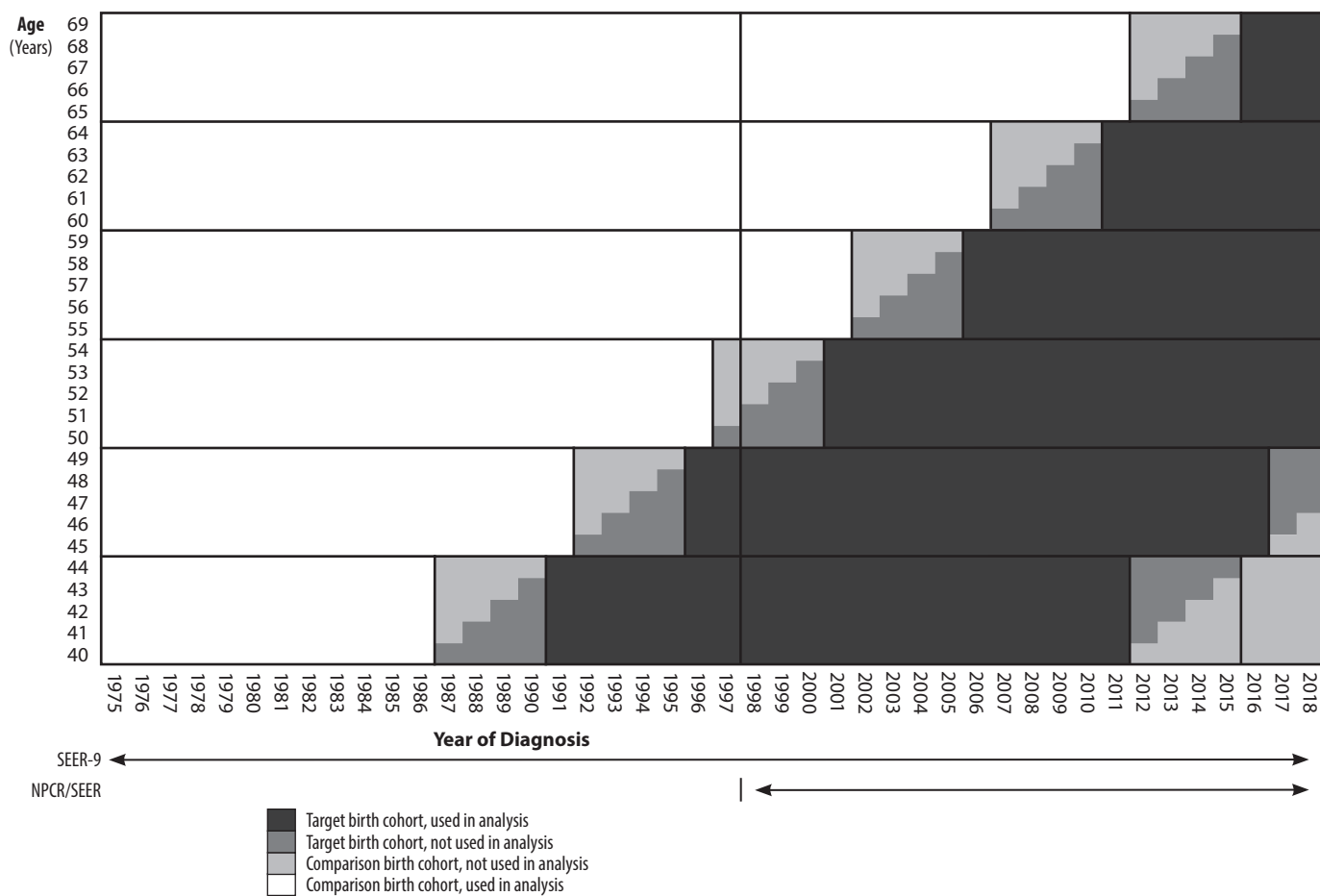

Supplement: Supplementary Figure 1 [file NIHMS1820146-supplement-Supplementary_Figure_1.pdf]
